# HIV- and AIDS-related knowledge and attitude of residents in border regions of Vietnam

**DOI:** 10.1186/s12954-019-0282-x

**Published:** 2019-02-07

**Authors:** Canh Dinh Hoang, Bach Xuan Tran, Manh Duc Pham, Long Hoang Nguyen, Ha Ngoc Do, Quan Hoang Vuong, Manh Tung Ho, Van Nhue Dam, Thu Trang Vuong, Hai Hong Nguyen, Vu Nguyen, Hai Quang Pham, Giang Hai Ha, Carl A. Latkin, Cyrus S. H. Ho, Roger C. M. Ho

**Affiliations:** 1 Vietnam Authority of HIV/AIDS Control, Ministry of Health, Hanoi, Vietnam; 2 Institute for Preventive Medicine and Public Health, Hanoi Medical University, Hanoi, Vietnam; 3 Bloomberg School of Public Health, Johns Hopkins University, Baltimore, MD USA; 4 Center of Excellence in Behavioral Medicine, Nguyen Tat Thanh University, Ho Chi Minh City, Vietnam; 5 Youth Research Institute, Ho Chi Minh Communist Youth Union, Hanoi, Vietnam; 6 Center for Interdisciplinary Social Research, Phenikaa University, Hanoi, Vietnam; 7 National Economics University, Hanoi, Vietnam; 8 Sciences Po Paris, Campus de Dijon, 21000 Dijon, France; 9 Department of Neurosurgery Spine-Surgery, Hanoi Medical University Hospital, Hanoi, Vietnam; 10 Institute for Global Health Innovations, Duy Tan University, Da Nang, Vietnam; 11 Department of Psychological Medicine, National University Hospital, Singapore, Singapore; 12 Department of Psychological Medicine, Yong Loo Lin School of Medicine, National University of Singapore, Singapore, Singapore

**Keywords:** Border zone, HIV/AIDS, Knowledge, Attitude, Vietnam

## Abstract

**Background:**

Residents in border areas are vulnerable to HIV/AIDS due to high rates of risk behaviors such as unprotected sexual practices or illicit drug use. Improving knowledge and attitude toward HIV/AIDS prevention and treatment are vital to diminish the burden of the HIV epidemic in this setting. However, evidence about this issue in Vietnam has been limited. This study aims to explore the knowledge and attitude toward HIV/AIDS among people in Vietnam border zones.

**Methods:**

We conducted a cross-sectional study in three border communes in Thanh Hoa province with 600 HIV(−) residents. Data about socio-demographic characteristics, general HIV knowledge, knowledge about prevention of mother-to-child transmission, treatment and care, HIV testing services, and attitude toward HIV/AIDS were collected. Multivariate Tobit regression was used to determine related factors with the knowledge and attitude.

**Results:**

The highest percentage of people having correct statements was for “HIV could be transmitted from mother to child” (98.2%), while the lowest percentage was for item “Know health facilities where HIV-positive people could register for care and check-up” (28.2%). People had the highest score in “Knowledge about HIV transmission routes” and the lowest score in “Knowledge about HIV/AIDS prevention measures”. Most of the people were not afraid of being exposed to HIV-positive individuals (66.0%), willing to buy goods from HIV-positive sellers (78.9%), and willing to take care of people living with HIV in their family (90.1%). Education, ethnic, marital status, occupations, and HIV/AIDS information sources were found to be associated with knowledge and attitude toward HIV/AIDS.

**Conclusions:**

The general knowledge and attitude on HIV/AIDS of residents were relatively good. Educational campaigns to improve knowledge and attitude toward PLWH, involving peer educators and local associations, are potential strategies for sustaining HIV intervention in this remote setting.

**Electronic supplementary material:**

The online version of this article (10.1186/s12954-019-0282-x) contains supplementary material, which is available to authorized users.

## Background

HIV/AIDS is contributing to a substantial global burden of diseases though a substantial progress in prevention and treatment has been made [1]. A recent report of UNAIDS estimated that in 2016, there were more than 1.8 million new HIV infections and 1.0 million deaths from AIDS-related causes [2]. Insufficient knowledge, attitude, and practice (KAP) toward HIV/AIDS prevention and treatment play a major role to elevate the risk of HIV/AIDS transmission in the community [3, 4], particularly most-at-risk populations such as men who have sex with men, female sex workers, and opiate-dependent individuals [5–7]. They are more likely to engage in HIV-related risk behaviors, or have knowledge about the risks but cannot translate into actions [8, 9]. As such, improving KAP about HIV/AIDS has been becoming a critical component in HIV/AIDS prevention and control strategies across nations, given its benefits in diminishing risk behaviors and increase safe practices as well as HIV testing uptake [10, 11].

Residents living in border zones are among the most vulnerable populations to HIV/AIDS transmission [12–14]. Evidence in different countries highlights the complexity of HIV-related issues in this setting. For instance, a study in the border area of Tanzania found that migration attributed to the high rates of unsafe sex practice, while a study in the cross-border of Mexico-the United States of America (USA) shows that opioid drug practice was common among people residing in this setting [13, 15]. Another study among female sex workers on the border between Brazil and French Guiana indicated a low rate of HIV/AIDS testing uptake due to a lack of proper services [16]. Studies in the border settings of Thailand revealed a low level of HIV-related KAP among residents in these areas [17, 18]. In a study in Iran, residents living in the border area reported that their knowledge mainly came from their individual experiences instead of health promotion programs [19]. Collectively, they pose a preferable condition for the escalation of HIV/AIDS epidemic, raising urgent needs of interventions to promote HIV-related KAP in order to prevent and reduce risk behaviors among residents in border zones [20, 21].

Vietnam has shared border areas with China, Laos, and Cambodia, creating a favorable environment for the illicit drug trade and trafficking [12, 22]. However, the implementation of HIV/AIDS researches and interventions in this setting is constrained because of difficult terrains, immigration, ethnic diversity, and crime [12, 22]. Previously, the governments of the four countries have had a strong commitment to reducing the risk of cross-border HIV transmission [22]. From 2013, a number of border provinces received foreign financial support to strengthen the health systems, build the capacities, and scale up the HIV services. Nonetheless, recently, these aids are speedily reduced because Vietnam becomes a low-middle-income country, leading to insufficient resources for these activities in this area. Therefore, it is important to provide more data about the HIV epidemic and the HIV/AIDS-related KAP of people living in border zones, contributing to the optimization of further interventions in these settings. This study aimed to explore the knowledge and attitude toward HIV/AIDS as well as identify associated factors with good knowledge in residents living in three Vietnamese border zones.

## Methods

### Study design and sampling method

A cross-sectional study was performed between September and October 2016 in Thanh Hoa, an HIV/AIDS epicenter in Northern Vietnam. Thanh Hoa has a long border with the Lao People’s Democratic Republic. This study was conducted in three border communes namely Bat Mon, Ten Tan, and Na Meo. Each commune has a border gate with the Lao People’s Democratic Republic.

A multistage sampling method was employed to recruit participants in this study. First, we listed all villages in three communes using data from local authorities and randomly chose six villages in each commune. Next, with these data, we developed a sampling frame that contained all households having people who matched the following criteria: (1) living in the study settings for more than 6 months and (2) aged from 15 to 49. In each village, we randomly selected 30–35 households by using a computer software (Stata version 13). Then, we visited selected households and randomly interviewed one person per household matching eligible criteria in order to reach 200 people/commune. With the support of local health workers, a total of 600 participants were included in the study (response rate = 100%).

### Measures and instruments

Face-to-face interviews were conducted with a structured questionnaire in 20–25 min by well-trained local health staffs. These interviewers were selected from local health staffs working at the commune health center in each setting. Before conducting the survey, they received a 3-day training session about sampling technique, the questionnaire, interview techniques, and other logistic concerns. Then, they participated in a pilot survey with a small sample size (20 residents) to test the questionnaire as well as to ensure that their interview technique was consistent with high quality. The pilot study was conducted to assure the appropriateness regarding cultural and language perspectives. For any questions that the residents did not understand the contents or the logical orders, we asked them to provide recommendations and then revised accordingly. After ensuring that people could understand clearly the questionnaire, we implemented the interview on a large scale (600 residents).

When meeting the eligible participants, we first introduced the purposes of the study, the benefits, drawbacks, and rights of participants if enrolling in the study and provided an information sheet for their consideration. Local health workers also helped us to explain the study to the residents in order to have residents’ agreement. Then, if they agreed to take part in the study, we asked them to provide written or verbal informed consents. We also invited them to go to a private room to ensure their confidentiality. They were allowed to withdraw anytime. All participants were informed that they would not receive any payment for the participation.

Information was investigated including socio-demographic characteristics (age, gender, education, occupation, ethnics, and marital status), whether they knew HIV/AIDS or not, and information sources for HIV/AIDS.

In this study, a series of questions about knowledge and attitude toward HIV/AIDS were built based on the monitoring indicators of Vietnam Administrative of HIV/AIDS Control (VAAC). The knowledge items included: (1) general HIV/AIDS knowledge, (2) knowledge about prevention of mother-to-child transmission, (3) knowledge about HIV treatment and care, and (4) knowledge HIV testing and counseling service. A correct response for each item was scored 1 point, and incorrect answer was scored 0 point. We also investigated our participants’ attitude regarding: “Being afraid of exposing to HIV-positive individuals,” “Buying goods from HIV-positive sellers,” “Willing to take care HIV-positive relatives in family,” “Agree with HIV-positive teacher continuing to teach,” and “Keeping secret of HIV-positive members in family”. The attitude score ranges from 0 to 5, with 5 as the score for people who responded in the way that proved positive attitude toward people living with HIV (PLWH) for all questions (see Additional file 1).

### Statistical analysis

Chi-squared and Mann-Whitney tests were used to identify the differences in socio-characteristics and attitude between males and females. The statistical significance was identified if a *p* value was under 0.05. In the current study, we used an exploratory factor analysis (EFA) to understand the construct validity of the knowledge questions. We also used a principal component analysis to extract appropriate factors (domains) with an eigenvalue of 1.3 and Orthogonal Varimax rotation to allocate items to suitable domains in order to improve the interpretability of our findings. The cut-off point was set at 0.4 for factor loading. Cronbach’s alpha was employed to measure the internal consistency of the questionnaire. After conducting EFA, three domains were detected namely “Knowledge about HIV/AIDS prevention measures,” “Knowledge about HIV-related service,” and “Knowledge about HIV transmission routes.” The values of Cronbach’s alpha were 0.68, 0.47, and 0.52, respectively. The score of each domain was calculated by summing scores of all items in this domain.

Scores of three domains as well as attitude score were transformed to 100-point scale to increase the comparability, with 0 point meaning “no correct answer/no positive attitude toward PLWH” and 100 points meaning “all answers are correct/completely positive attitude toward PLWH”. Because these data about scores were censored (lower limit = 0; upper limit = 100), we utilized multivariate Tobit regression to identify the factors related to good knowledge and positive attitude. Forward stepwise selection strategy was utilized to build the final regression models.

## Results

Table 1 shows that most of the respondents were male (59.7%) with the mean age of 32.6 years old (SD = 8.2). The prevalence of people who had a secondary education was the highest among participants (48.5%). The majority of participants were Thai ethnic (94.5%) and living with spouse/partner (93.5%). Farmer and forestry workers were the dominant jobs among our respondents (78.2%). No difference was found between males and females regarding age, education, ethnic, and marital status (*p* > 0.05). The proportion of freelancer—those who had to do varied kinds of the job for money—in males (21.0%) was significantly higher than that in females (13.6%) (*p* < 0.05).

**Table 1:** Demographic characteristics of respondents (*n* = 600)

Characteristics	Male		Female		Total		*p* value
	*n*	%	*n*	%	*n*	%	
Total	358	59.7	242	40.3	600	100.0	
Education attainment							
Illiterate	10	2.8	7	2.9	17	2.8	0.82
Elementary school	65	18.2	51	21.1	116	19.3	
Secondary school	176	49.2	115	47.5	291	48.6	
High school	96	26.8	59	24.4	155	25.8	
> High school vocational training/college	11	3.1	10	4.1	21	3.5	
Ethnic							
Thai	335	93.6	232	95.9	567	94.5	0.23
Others	23	6.4	10	4.1	33	5.5	
Marital status							
Live with spouse/partner	332	92.7	229	94.6	561	93.5	0.36
Others	26	7.3	13	5.4	39	6.5	
Occupation							
Farmer, a forestry worker	272	76.0	197	81.4	469	78.2	0.04
Freelancer	75	21.0	33	13.6	108	18.0	
Other	11	3.0	12	5.0	23	3.8	
	Mean	SD	Mean	SD	Mean	SD	
Age	32.9	8.2	32.1	8.2	32.6	8.2	0.25

Among our respondents, there was 99.7% who knew HIV/AIDS before and health workers were the most common information source of HIV/AIDS (88.6%), following by mass media (63.0%) and local loudspeakers (52.3%). No difference was found regarding information sources among males and females (*p* > 0.05) (Table 2).

**Table 2:** Information source about HIV/AIDS (*n* = 600)

Characteristics	Male		Female		Total		*p* value
	*n*	%	*n*	%	*n*	%	
Know about HIV/AIDS	357	99.7	241	99.6	598	99.7	0.99
Information source							
Spouse/partner	31	8.7	11	4.6	42	7.0	0.05
Friend/relative	55	15.4	25	10.4	80	13.4	0.08
Health worker	323	90.5	207	85.9	530	88.6	0.08
Peer educators	20	5.6	14	5.8	34	5.7	0.92
Mass media	229	64.2	148	61.4	377	63.0	0.50
Local loudspeakers	179	50.1	134	55.6	313	52.3	0.19

Table 3 reveals the results of the EFA. The highest percentage of people having a correct answer was 99.7% for item “Know the places where could have HIV testing”, while the lowest percentage was 28.2% for item “Know health facilities where HIV-positive people could register for care and check-up”. People had the highest score in “Knowledge about HIV transmission routes” and the lowest score in “Knowledge about HIV/AIDS prevention measures”.

**Table 3:** Knowledge about HIV/AIDS and psychometric properties of measures (*n* = 598)

Items	Having correct answers		Knowledge about HIV/AIDS prevention measures	Knowledge about HIV-related service	Knowledge about HIV transmission routes
	*n*	%			
Know the places where could have HIV testing	596	99.7		0.65	
Know HIV could be transmitted from mother to children	587	98.2	0.40		
Sharing needles when injected drug could transmit HIV	585	97.8			0.60
Using condom can prevent HIV	580	97.0	0.39		
Know the place where could receive/buy a clean needle	560	93.7		0.54	
Know the place to take or buy a condom	555	92.8		0.54	
Having meals with HIV(+) people could not be infected HIV	543	90.8			0.57
Mosquitoes cannot transmit HIV from person to person	541	90.5			0.36
A healthy-looking person could be HIV positive	477	79.8			0.36
Know blood-related exposures could cause HIV transmission	417	69.5			0.38
Know sexual risk behaviors could cause HIV transmission	323	53.8			0.40
Benefits of using clean needles	290	48.3	0.75		
Know necessary preparations when taking care HIV(+) people with illness	250	41.7	0.67		
Know the benefits of using a condom	225	37.5	0.84		
Know health facilities where HIV(+) people could register for care and check-up	169	28.2		0.79	
Cronbach’s alpha			0.68	0.47	0.52
Mean			53.3	64.6	78.6
SD			18.4	26.1	16.9

Table 4 reveals that most of our sample were not afraid of exposing to HIV-positive individuals (66.0%), willing to buy goods from HIV-positive sellers (78.9%), and take care of HIV-positive relatives in the family (90.1%). The majority of our respondents stated that a teacher with HIV-positive should continue to teach other people (81.8%). However, only 44.2% kept the secret of HIV-positive members of the family. There was no difference between males and females regarding attitude items and attitude score (*p* > 0.05).

**Table 4:** Attitude toward HIV/AIDS among respondents (*n* = 598)

Characteristics	Male		Female		Total		*p* value
	*n*	%	*n*	%	*n*	%	
Buying goods from HIV(+) sellers							
Yes	278	78.1	193	80.1	471	78.9	0.56
No	78	21.9	48	19.9	126	21.1	
Willing to take care HIV(+) relatives in the family							
Yes	317	88.8	222	92.1	539	90.1	0.18
No	40	11.2	19	7.9	59	9.9	
A teacher with HIV(+) should continue to teach other people							
Yes	285	79.8	204	84.7	489	81.8	0.14
No	72	20.2	37	15.4	109	18.2	
Keeping secret of HIV(+) members in the family							
Yes	161	45.1	103	42.7	264	44.2	0.57
No	196	54.9	138	57.3	334	55.8	
Afraid of being exposed to HIV(+) individuals							
Very afraid	36	10.1	19	7.9	55	9.2	0.16
Afraid	96	26.9	52	21.6	148	24.8	
Not afraid	225	63.0	170	70.5	395	66.0	
Reasons for afraid of being exposed to HIV(+) people (*n* = 203)							
Fear of HIV transmission	124	93.9	67	94.4	191	94.0	0.91
Disrespect HIV(+) people	3	2.3	2	2.8	5	2.5	
Others	5	3.8	2	2.8	7	3.5	
	Mean	SD	Mean	SD	Mean	SD	
Attitude score	70.7	26.8	73.7	23.9	71.9	25.7	0.28

Results of regression models are shown in Table 5**.** People living with spouse/partner and who were Thai ethnic had a higher score in “Knowledge about HIV transmission” and “Knowledge about HIV-related service”. Respondents who were freelancers also had a higher score in “Knowledge about HIV-related service”. Meanwhile, higher age, higher education, and farmer/forestry workers were found to be associated with a higher score in “Knowledge about HIV/AIDS prevention measures”. People who were freelancers had a lower attitude score compared to white-collar workers (Coef. = − 9.8; 95%CI = − 16.5; − 3.0).

**Table 5:** Factors associated with knowledge and attitude about HIV/AIDS (*n* = 600)

	Knowledge about HIV transmission		Knowledge about HIV/AIDS prevention measures		Knowledge about HIV-related service		Attitude toward HIV/AIDS	
	Coef.	95%CI	Coef.	95%CI	Coef.	95%CI	Coef.	95%CI
Age			0.4**	0.1; 0.7				
Gender (female vs male)			− 4.2*	− 8.8; 0.4	− 2.3	− 5.6; 1.0	− 3.7	− 8.9; 1.4
Education (vs illiterate)								
Elementary school	− 5.1	− 14.1; 3.9	6.3	−7.5; 20.2	1.0	− 9.1; 11.0		
Secondary school	− 1.7	− 10.4; 7.1	21.2***	7.6; 34.6	5.8	− 3.9; 15.6		
High school	− 2.5	− 11.6; 6.6	26.9***	12.6; 41.1	9.8*	− 0.5; 20.0		
Vocational training/college	4.0	− 8.0; 16.0	21.81**	2.9; 40.8	− 7.6	− 21.2; 6.0		
≥ University	21.5*	− 1.5; 44.5	35.59**	0.9; 70.3	− 0.14	− 25.1; 24.8		
Ethnic (vs Muong)								
Thai	18.8***	9.1; 28.6	10.56	− 4.9; 26.0	11.4**	0.5; 22.4		
Others	0.3	− 12.0; 12.7	− 1.27	− 20.6; 18.1	5.0	− 8.8; 18.9	− 12.4*	− 26.1; 1.3
Marital status (vs single)								
Live with spouse/partner	16.8***	9.8; 23.7	9.79*	− 1.3; 20.9	9.7**	1.8; 17.6		
Divorce/widow	4.1	− 9.6; 17.8	7.06	− 14.5; 28.6	− 6.2	− 21.7; 9.3		
Occupation (vs White-collar workers)								
Farmer, a forestry worker	0.5	− 7.7; 8.7	15.21**	2.3; 28.1	6.0	− 3.4; 15.4		
Freelancer	7.5*	− 0.9; 15.9	9.70	− 3.5; 22.9	11.9**	2.2; 21.6	− 9.8***	− 16.5; − 3.0
Unemployed	13.1	− 9.5; 35.6	− 11.80	− 45.5; 21.9	− 18.1	− 42.5; 6.3		
Information sources (yes vs no)								
Spouse/partner	8.8***	3.1; 14.6	− 15.66***	− 25.7; − 5.6				
Friend/relative			− 6.06	− 13.6; 1.5				
Health worker	4.9**	0.1; 9.6			− 6.9**	− 12.5; − 1.3	− 9.2	− 20.4; 2.0
Peer educators			18.32***	7.8; 28.8	8.0**	0.9; 15.1	9.2**	0.5;17.8
Mass media	4.7***	1.5; 7.9	11.95***	6.9; 17.0	15.7***	12.1; 19.2		
Local loudspeakers	6.4***	3.2; 9.5	29.18***	24.1; 34.3	7.7***	4.2; 11.3	22.0***	16.6; 27.5

*p < 0.1;**p < 0.05;***p < 0.01

People receiving information from peer educators, local loudspeakers, and mass media had higher knowledge and attitude scores than those who did not. Hearing information from spouse/partner and health workers was positively associated with the higher score in “Knowledge about HIV transmission”. Nonetheless, hearing from spouse/partners was related to a lower score in “Knowledge about HIV/AIDS prevention measures”. People getting information from health workers had a lower score in “Knowledge about HIV-related service”. Notably, knowing information from peer educators or local loudspeakers was associated with higher attitude score.

## Discussion

This study contributes to the existing literature on knowledge and attitude of HIV/AIDS among people in some Vietnam border zones. In this study, we found a moderately good knowledge and attitude on HIV/AIDS of people living in border settings; however, knowledge on safe practice in caring for patients and availability of health care services should be improved. These insights are necessary to inform the development of HIV interventions in this disadvantaged setting.

In the current study, our respondents have shown a moderately high level of HIV knowledge: correctly identifying needles sharing as one transmission route while also exhibiting awareness of some misconceptions (transmission via mosquitoes, healthy-looking person, etc.). This is comparable to that found in other populations such as HIV-positive patients taking antiretroviral therapy or young individuals [23, 24]. However, our findings indicated a gap in the knowledge about transmission via sexual behaviors or blood-related routes. In addition, a lack of knowledge about HIV prevention measures and HIV-related services among participants has also been found. Our results were even lower compared to that of the Vietnamese general population, which indicated that more than 45% of young people knew the methods of preventing HIV transmission [25].

The study found that 34% of participants reported that they were “afraid” and “very afraid” to come in contact with PLWHA. The fear of HIV transmission (94.1%) was stated as the main reason. A similar reason was stated by a lower proportion of participants (58.1%) in a study by Hoang et al. concerning the border communes of [26]. Meanwhile, the proportion of people who were willing to buy goods from HIV-positive sellers or take care of HIV-positive family member were found to be higher than these rates in the study among Vietnamese general population conducted by Vu et al. (using Audio Computer-Assisted Self-Interview (ACASI)) [27] or in Survey Assessment of Vietnamese Youth 2 (SAVY2) [24]. Vu et al. suggested that differences between findings of these studies may be due to the application of different interview methods, in particular in face-to-face interviews people may less likely to tell their true opinions about discrimination, stigma, or responsibility to family members [27]. Otherwise, the finding in this study may positively suggest that our sample in border zones had less discrimination and stigmatization toward HIV-positive individuals compared to the general population [28].

These are several important factors that should be considered for planning or implementing interventions for the population in this region. Education was found to be positively associated with knowledge on HIV/AIDS. Moreover, in the border regions, there are different ethnic groups with their own community languages, such as Thai and Muong people. Therefore, communication campaigns should take into account the cultural diversity in designing messages and channels. Furthermore, respondents who have higher levels of knowledge or attitude were more likely to receive information from peer educators, mass media, or local loudspeakers. In this remote setting, we observed that health workers and mass media campaigns are major HIV-related information sources. Although peer-educators were very helpful in referring to people who were at-risk or needed care to appropriate health services [29–32], their involvement in communication activities was limited.

Implications of the study findings are drawn to inform the design of HIV prevention and harm reduction campaigns and scale-up of care and treatment services. First of all, as a medium level of HIV-related knowledge was found among residents in the border areas, educational campaigns should be assured to improve this situation. Second, besides mass media educational campaigns, peer-educators should be promoted. This includes the potential involvement of youth, women, or farmers unions in the local context. Finally, different levels of knowledge on HIV/AIDS between different ethnic groups suggest that more socio-cultural sensitive considerations should be included in the development of HIV interventions.

Although evidence in the context of border region was very limited and this is one of the very first large surveys in Vietnam, there were some limitations of the study that should be presented. First, recall bias could occur due to self-reported approach. Second, we only had a moderate reliability of the instrument that possibly affects the validity of the measurement. The questionnaire was built based on monitoring indicators issued by the Vietnam Administrative of HIV/AIDS Control (VAAC) which were validated for the Vietnam context. We suspected the level of reliability seen might be caused by the sociocultural, in many cases language differences of communities in the border regions, which future studies should consider more carefully in designing the study instrument. Third, despite using the multistage sampling method, our results might not represent the knowledge and attitude of the whole residents in other border zones. Therefore, further large-scale studies should be conducted in order to find out more representative results in the future.

## Conclusion

In this border region, the general knowledge and attitude on HIV/AIDS of residents were relatively good. Educational campaigns to improve knowledge and attitude toward PLWH, involving peer-educators and local associations, are potential strategies for sustaining HIV intervention in this remote setting.

## Additional file

Additional file 1: Questionnaire of the study. (DOCX 27 kb)

## Abbreviations

KAP: Knowledge, attitude, and practice
PLWH: People living with HIV
