# Supplementary material for: HIV- and AIDS-related knowledge and attitude of residents in border regions of Vietnam
Source: Harm Reduct J. 2019 Feb 7;16:11. doi: 10.1186/s12954-019-0282-x (PMC6367780; doi:10.1186/s12954-019-0282-x)
Supplement: Supplementary file 1 — Questionnaire of the study. (DOCX 27 kb) [file 12954_2019_282_MOESM1_ESM.docx]

**SURVEY QUESTIONNAIRE**

**PART 1. GENERAL INFORMATION**

| **Order** | **Question** | **Answer** | **Code** |
| --- | --- | --- | --- |
| C.101 | Gender | Male  Female | 1  2 |
| C.102 | Date of birth | Year | \|__\|__\|__\|__\| |
| C.103 | What is your current level of education? | Illiterate  Elementary school  Secondary school  High school  Vocational training/College  University/Postgraduate | 1  2  3  4  5  6 |
| C.104 | What is your ethnic? | Kinh  Muong  Thai  H’Mong  Other (specify):….……………….. | 1  2  3  4  5 |
| C.105 | What is your current marital status? | Single  Having spouse  Divorce  Widow  Separate  Having partner | 1  2  3  4  5  6 |
| C.106 | What is your main occupation? | White-collar worker  Farmer, forestry worker  Freelancer  Unemployed | 1  2  3  4 |

**PART II. KNOWLEDGE AND ATTITUDE REGARDING HIV/AIDS**

| **Order** | **Question** | **Answer** | **Code** |
| --- | --- | --- | --- |
| C.201 | Have you ever heard about HIV/AIDS | Yes  No | 1  2 |
| C.202 | **If yes,** Could you please list some sources of information that you obtained from? | Spouse/Partner  Friend/Relative  Health worker  Peer educators  Mass media  Local loudspeakers | 1 |
|  |  |  | 2 |
|  |  |  | 3 |
|  |  |  | 4 |
|  |  |  | 5 |
|  |  |  | 6 |
|  | **Knowledge about HIV/AIDS** |  |  |
| C.203 | Can mosquitous’s bite transmit HIV? | Yes  No  Don’t know | 1  2  3 |
| C.204 | Can using condom prevent the transmission of HIV? | Yes  No  Don’t know | 1  2  3 |
| C.205 | Can a healthy-looking person be HIV positive? | Yes  No  Don’t know | 1  2  3 |
| C.206 | Can eating with HIV(+) person transmit HIV? | Yes  No  Don’t know | 1  2  3 |
| C.207 | Can sharing needles when injected drug transmit HIV? | Yes  No  Don’t know | 1  2  3 |
|  | **Attitude toward HIV/AIDS** |  |  |
| C.208 | Are you afraid of exposing to HIV (+) individuals? | Very afraid  Afraid  Not be afraid | 1  2  3=>**C.211** |
| C.209 | Could you please tell us about the reasons for being afraid of exposing to HIV(+) people? | Fear of HIV transmission  Disrespect HIV(+) people  Others (specify):…………………… | 1  2  3 |
| C.210 | Would you want to buy goods from HIV (+) sellers? | Yes  No | 1  2 |
| C.211 | Are you willing to take care HIV (+) individuals in family? | Yes  No | 1  2 |
| C.212 | Do you think that teacher with HIV (+) should continue to teach other people? | Yes  No | 1  2 |
| C.213 | Are you willing to keep secret of HIV (+) members in family | Yes  No | 1  2 |
|  | **Knowledge about HIV-related risk behaviors** |  |  |
| C.214 | In your opinion, which following activities are high risk behaviors for HIV transmission? | Having sexual intercourse with sex workers without condom  Having sexual intercourse with people suffering STI without condom  Having sexual intercourse with HIV(+) people without condom  HIV(+) people want to have a child  All of options above  Don’t know | 1  2  3  4  5  6 |
| C.215 | In your opinion, which following activities are high risk behaviors for HIV transmission via the blood route? | Inject illegal drug  Sharing needles with people who injected drug  Receiving blood transfusion without screening HIV  Conducting first aids to people who are bleeding without protective gears  Not wearing gloves when exposing to blood and fluid of patients  All of options above  Don’t know | 1  2  3  4  5  6  7 |
| C.216 | In your opinion, what is the benefit of condom? | Prevent pregnancy  Prevent STI transmission  Prevent HIV transmission  Don’t know | 1  2  3  4 |
| C.217 | Do you know where you can buy condoms? | Yes  No | 1  2 |
| C.218 | In your opinion, what is the benefit of clean syringes/ needles? | Prevent blood-related infections  Prevent HIV via sharing needles  Don’t know | 1  2  3 |
| C.219 | Do you know where you can buy clean syringes/needles? | Yes  No | 1  2 |

**PART III. KNOWLEDGE ABOUT MOTHER-TO-CHILD TRANSMISSION**

| **Order** | **Question** | **Answer** | **Code** |
| --- | --- | --- | --- |
| C.301 | Can HIV transmit from mother to children? | Yes  No | 1  2**=>C.401** |
| C.302 | How can HIV be transmitted from mother to children?  *(Nhiều lựa chọn)* | During pregnancy  During giving birth  When breast feeding  All of options above  Don’t know | 1  2  3  4  5 |
| C.303 | In your opinion, is there any drugs to prevent the HIV transmission from mother to children? | Yes  No  Don’t know | 1  2  3 |

**PART IV. KNOWLEDGE ABOUT HIV/AIDS CARE AND TREATMENT**

| **Order** | **Question** | **Answer** | **Code** |
| --- | --- | --- | --- |
| C.401 | In your opinion, what do you need to prepare when taking care of HIV(+) patients? | Always wear gloves when washing clothes, bed if blood is sticky  Always wear a mask when an infected person coughs, or gobs  Do not know | 1  2  3 |
| C.402 | In your opinion, where can HIV(+) register to treatment and care? | General hospital  Provincial/District Health Center  Outpatient clinic  Commune health center  Do not know | 1  2  3  4  5 |

**PART V. KNOWLEDGE ABOUT HIV TESTING AND COUNSELING**

| **Order** | **Question** | **Answer** | **Code** |
| --- | --- | --- | --- |
| C.501 | Do you know where people can visit for HIV testing? | Yes  No | 1  2**=>C.503** |
| C.502 | Where is it? | HIV/AIDS Prevention Center  District Health Center  General Hospital  Commune Health Center  HIV Counseling and Testing Clinic  Private clinic  Don’t know | 1  2  3  4  5  6  7 |
| C.503 | In your opinion, why do we need to test HIV?  *(Một lựa chọn)* | Health examination  To know whether infect HIV or not  To have early treatment  To know the way to reduce the risk of HIV transmission to other people | 1  2  3  4 |
